# Infectious Diseases and Vaccination Strategies: How to Protect the “Unprotectable”?

**DOI:** 10.5402/2013/765354

**Published:** 2013-04-03

**Authors:** Elena Bozzola, Mauro Bozzola, Valeria Calcaterra, Salvatore Barberi, Alberto Villani

**Affiliations:** ^1^Department of Pediatrics, Pediatric and Infectious Disease Unit, Bambino Gesù Children's Hospital, 00100 Rome, Italy; ^2^Department of Internal Medicine, University of Pavia, 27100 Pavia, Italy; ^3^Department of Pediatrics, San Paolo Hospital, University of Milan, 20121 Milan, Italy

## Abstract

*Introduction*. The circulation of infectious diseases puts small infants too young to be vaccinated at risk of morbidity and mortality, often requiring prolonged hospitalization. *Material and Methods*. We have reviewed the medical records of children not eligible for vaccination because of age, admitted to hospital for pertussis, measles, or varicella from February 1, 2010, till February 1, 2012. *Results*. Of the case records scrutinized, 21 were hospitalized for pertussis, 18 for measles, and 32 for varicella. Out of them, 42%, 66%, and 78% diagnosed with, respectively, pertussis, measles, and varicella had a complicated course of the disease. *Discussion*. To avoid infectious disease circulation, childhood immunization strategies should be adopted, such as vaccination of healthcare givers, adult household contacts, and parents planning to have, or who have had, a newborn baby.

## 1. Introduction

Since the days of Jenner and Pasteur, inducing an immune response to infectious diseases by the way of vaccination has become a widely applied intervention to keep people healthy. Globally, the population coverage of vaccination programs has expanded so that immunization has served to eradicate potential fatal diseases, such as smallpox [1]. Moreover, vaccination has stickling reduced the morbidity and mortality due to childhood infectious diseases, such as pertussis and measles, in developed countries. In Italy the schedule for pertussis vaccination consists of a course of a free-of-charge vaccination by 3 months of age and a booster dose at 5-6 years, in combination with tetanus and diphtheria. Measles-mumps-rubella vaccine is currently offered free of charge in a two-dose routine immunization program at 12–15 months and at 5-6 years of age, respectively [2, 3]. However, in the last years, a resurgence of both pertussis and measles has been experienced. The outbreaks occurred predominantly among older children and young adults who had not been vaccinated. As well as in other countries, in Italy, pertussis and measles are still circulating and can run a severe course in young infants, requiring hospitalization [4]. As for varicella, in 1995, in the United States the vaccine was first introduced as part of the routine childhood vaccination program. Since then, clinical trials and postmarketing surveillance studies have shown that the vaccine is effective and safe, so that new prospects of the prevention of varicella have been opened. In Italy, varicella vaccine is available since 2001 but is not included in the routine childhood vaccination schedule of most regions and is not free of charge. The childhood immunization schedule consists of a single dose at 12 months of age and of a catch-up vaccination for susceptible children aged 12 years. The immunization may be offered in association with measles-mumps-rubella vaccine.

## 2. Aim of the Study

The aim of our study was to evaluate the morbidity and mortality of pertussis, measles, and varicella in infants too young to be vaccinated. We also discussed possible preventive policies.

## 3. Material and Methods

We reviewed the medical records of children admitted to Bambino Gesù Children's Hospital, in Rome, Italy, between February 1, 2010, till February 1, 2012. Bambino Gesù Children's Hospital has nearly 450 pediatric beds and serves as a third-level pediatric referral centre for Rome, the Italian capital. The inclusion criteria were

- unvaccinated children younger than 3 months of age diagnosed with pertussis,
- unvaccinated children younger than 15 months diagnosed with measles,
- unvaccinated children younger than 12 months of age diagnosed with varicella.

The diagnosis of varicella was based on clinical evidence of characteristic skin lesions in varying stages of development and resolution. Laboratory diagnosis was required just in 2 patients. In these doubtful cases, serological tests measured specific immunoglobulin M (IgM) in one serum specimen, confirming varicella infection. As for pertussis, the clinical diagnosis was confirmed in all cases by specific polymerase chain reaction (PCR) on a nasopharyngeal sample. In case of measles exanthema, both PCR and IgM validated the diagnosis in all patients.

## 4. Results

Of the case records scrutinized, 21 unvaccinated infants younger than 3 months of age (mean age 54 days) were hospitalized for pertussis. Out of them, 19% experienced apnea and 23% cyanosis. The mean hospitalization was of 14 days. In one case, due to the compromised clinical condition, the hospitalization was of 59 days. Fortunately, none of the infants in the study died or had severe invalidating complications, such as encephalopathy or intracranial bleeding. 

As for measles, 18 young unvaccinated children under 15 months were hospitalized during the study period. The mean age was of 7 months. The mean duration of hospitalization was of 6 days. In 66,6% a complicated disease was diagnosed. Among the most frequent observed complications there were dehydration in 4 cases (22%) and pneumonia in 6 (33%). None of the patients died or had disabling sequelae after discharge.

Finally, during the study period, 32 young unvaccinated children less than 12 months (mean age 8 months) were admitted for varicella. Out of them, 78% had a complicated course and remained at hospital for a mean time of 8 days. The most frequent complications were anemia and/or neutropenia (28,1%), impetigo (15,6%), pneumonia and/or bronchitis (15,6%), and dehydration (9,3%). None of the children died or presented with after-discharge sequelae.

## 5. Discussion

The failure of immunization coverage has to be considered as the primary reason for the transmission of pertussis and measles to young infants not eligible for vaccination because of age. The high circulation of pertussis and measles puts small infants too young to be vaccinated at risk of morbidity and mortality [4]. Infected infants frequently require hospitalization or develop complications, and the youngest have the highest risk of fatal disease [5]. In our study, among the case records scrutinized, 42% of the infants hospitalized for pertussis and 66% of those for measles had a complicated course of the disease. Moreover, infected infants had a prolonged hospitalization, respectively, of 14 and of 6 days. Consequently, to avoid infectious disease circulation, childhood immunization has to be considered as a major preventive health strategy [1]. Moreover, a number of preventive measures should be implemented to protect infants too young to be vaccinated and to reduce hospitalization and complication rates.

Regarding pertussis prevention, a wide range of control measures should be helpful. First the vaccination schedule could start at 6 weeks of age, which theoretically would prevent severe course in infants [6]. In fact some authors speculate that protection against severe pertussis can be achieved after one dose of vaccination [4, 7, 8]. Moreover, duration of hospitalization, which can be considered as a marker of severity, is significantly lower among infants who had received at least one dose of vaccine [4]. Second, vaccination of adult household contacts could be considered in vaccination planning [9, 10]. Multinational studies attempted to clarify the sources of pertussis infection in young infants and concluded that household caregivers were the source in most cases [4, 9, 10]. Adolescent and adults, as well as unvaccinated children, are likely to introduce pertussis in household. In a multicentre, prospective study, it was estimated that a single dose of vaccine in adolescents and adults, between the ages of 15 and 65 years, gave a protective efficacy of 92% (95% CI: 32–99%) for pertussis, confirmed by culture, PCR, or serologic assay [11, 12]. Moreover, a recent study evidenced that immunizing adolescents with a pertussis booster is the most economical and easiest-to-implement strategy to protect infants and should provide significant health and economic benefits [13, 14]. Unfortunately, in Italy, according to the ICONA 2008 survey, the vaccine immunization coverage in adolescents was 45.6% with three doses, 26.7% with four doses, and only 14.1%, with five doses, respectively. To improve vaccination coverage among adolescents, healthcare providers should take advantage of every healthcare visit as an opportunity to evaluate vaccination status and administer vaccines when needed. It is time to encourage providers to vaccinate adolescents on every possible occasion. Missed opportunities continue to be a major problem in meeting the targeted objectives. The experience in Australia shows that a broad school-based catch-up program followed by immunization of school entrants may be the most favorable strategy for the implementation of an adolescent vaccination program. A pertussis vaccination catch-up program could have an impact on herd immunity and on the incidence of disease among infants [15]. Vaccination of parents could substantially reduce the burden of infant pertussis [9, 10]. Immunizing parents planning to have, or who have had, a newborn baby with a booster dose may help to protect infants even in the first weeks of life [16–18]. In fact adults may experience waning immunity even if they completed a primary course of vaccination [19]. In a German study, measurable levels of antibodies against pertussis were found in just 37% of pregnant women [20]. Recent studies established that the lack of maternal immunity is one reason for pertussis susceptibility in very young infants [20, 21]. Newborns from mothers who received vaccination during pregnancy had significantly higher concentrations of antibodies, when compared to newborns born from mothers who did not receive the immunization during pregnancy [22]. Maternal vaccination would prevent infant infections from delivery until immunity is induced by active immunization. Besides, pregnancy is not a contraindication to pertussis immunization [16]. A concern that has been raised is the possible interference of pertussis-specific passive antibodies in infants who receive active immunization with pertussis. Some studies have suggested that the presence of maternal pertussis antibodies, as a consequence of vaccination during pregnancy, can have a negative effect on vaccine response of their children after administration of the vaccine. The inhibition of active pertussis-specific antibody production in those infants is referred to as “blunting.” The clinical importance of blunting is not clear, but it is merely a temporary effect, because passive maternal antibodies decline rapidly, within the first six months of an infant's life (half-life of approximately six weeks in infant sera) [23–25].

As for measles, to prevent transmission among infants too young to be immunized, a wide range of control measures should be implemented, such as engaging in social mobilization and advocacy for immunization among the household contacts and the healthcare providers. This is particularly relevant for healthcare personnel who is at increased risk for acquiring measles which can be then transmitted to susceptible contacts. In fact, primary protection against infectious diseases at birth provided by maternal antibodies endures for few months. Then, measles-specific maternal antibodies decline gradually during the first year of life with the development of the infants' own immune system. Consequently, the timing of the vaccination should be carefully determined, and the interval between vaccination and the loss of maternally derived antibodies should be minimized to protect children from infection with measles [26, 27]. Adolescents and adults are at the present time identified as the primary source of infection to susceptible and unprotected infants. Eliminating measles will require that the demand for vaccination should be increased in order to achieve and sustain >95% coverage with two doses of measles containing vaccine and that surveillance should be strengthened to ensure the timely identification of cases and outbreaks. Implementing active immunization activities, including offering free vaccination to people who take care of infants as well as to parents planning to have a new baby, should also be considered. An intriguing and very important social phenomenon is the parents' worldwide acceptance of childhood immunization [1]. The principal factors contributing to the decreased demand for measles vaccine include hesitancy to be vaccinated due to a lack of knowledge of the seriousness of the disease, skepticism about the vaccination benefits, and increased fear of adverse events following immunization. Consequently, in order to implement vaccination coverage, misinformation regarding vaccines must be addressed promptly. Physicians represent the best opportunity to influence the vaccine-hesitant people, providing accurate information about both risks and benefits. In fact, most parents trust their primary care providers and look to them for information and advice. Demonstrating a willingness to listen respectfully, encouraging questions, and acknowledging parental concerns are essential elements of this strategy. Effective communication requires understanding parents' reasons for resisting vaccination as well as the discussion of risks associated with remaining unvaccinated [28]. Finally, varicella has been described as a serious health condition in infants younger than 12 months, who are at increased risk for varicella-related hospitalization and death compared with older children. During our study period, 32 unvaccinated infants because of age had been hospitalized for varicella and 78% of them experienced a complicated disease. The mean hospitalization was of 7 days. To avoid varicella circulation, childhood immunization has to be considered as a major preventive health strategy. High rates of varicella vaccination in the community can also protect individuals who cannot be vaccinated because of age, avoiding hospitalization. By the way, a previous study demonstrated the great indirect benefits of the varicella vaccination program in protecting infants through lowered risk of exposure as a result of high population immunity [29, 30]. Consequently, preventive measures, such as catch-up varicella among older age group and a selected immunization of child care workers and parents of newborns, should be implemented to protect infants who are not eligible for varicella vaccination because of age. Vaccination against varicella should also be recommended for healthcare workers because they are at increased risk for acquiring and transmitting such disease to susceptible contacts.

Prevention of the disease has improved markedly due to several reasons, and especially to the availability of new vaccines and new combination vaccines. Nevertheless, immunization coverage is still low. Where varicella immunization programs are not in place, the incidence in children is high and is thus a significant economic burden for the community, mainly due to the loss of workdays for parents. Of particular concern is the disappointingly low uptake of vaccine in Italy, as the coverage is approximately about 5%. Varicella vaccine coverage depends strongly on the acceptance of the vaccination by parents. Parents are free to decide on their child's vaccinations and vaccination is not mandatory for daycare or school admission. Thus, recommendations by paediatricians may be useful in order to advice immunization. Nevertheless, paediatricians may underestimate both the potential risk of the disease and the economic burden for the community due to herd immunity and a reduced economic burden, thus recommending vaccination only for special risk groups. Consequently, difficulties may be found to protect susceptible infants, especially those too young to be immunized. Parents may consider the potential profit for the community as less important than the individual risk to their child from potential unintentional side effects of vaccination. Hence, high coverage levels might be difficult to achieve. Differently from pertussis, for measles and varicella, the pace of pathogenesis may be sufficiently slow to allow immune memory responses to intervene and prevent the important, disease-causing second viremic phase in most individuals. Consequently, booster doses of measles and varicella vaccines are not currently recommended, even if waning immunity has been raised as a concern [31–35]. Additional doses of these vaccines have been suggested to produce immunity among a relatively small cohort of individuals who fail to respond to primary vaccination [19].

## 6. Conclusion

In conclusion, in order to protect infants, a single approach may not be sufficient, and multiple immunization strategies for enhancing vaccination should become a public health priority and should be applied in a concerted mode.
